# A DNA-based real-time PCR assay for robust growth quantification of the bacterial pathogen *Pseudomonas syringae* on *Arabidopsis thaliana*

**DOI:** 10.1186/s13007-016-0149-z

**Published:** 2016-11-21

**Authors:** Annegret Ross, Imre E. Somssich

**Affiliations:** Department for Plant-Microbe Interactions, Max-Planck-Institute for Plant Breeding Research, Carl-von-Linné Weg 10, 50829 Cologne, Germany

**Keywords:** *Pst* DC3000, Plate counting assay, qRT-PCR

## Abstract

**Background:**

The interaction of *Pseudomonas syringae* with Arabidopsis is one of the most commonly used systems to study various bacterial—host interrelationships. Currently, most studies are based on the growth quantification of the pathogen to characterize resistance or virulence targets. However, the standard available method for determining bacterial proliferation in planta is laborious and has several limitations.

**Results:**

Here we present an alternative robust approach, which is based on the quantification of bacterial DNA by real-time PCR. We directly compared this assay with the routinely used plate counting method to access bacterial titers in a number of well described Arabidopsis mutants.

**Conclusions:**

These studies showed that the DNA-based technique is highly reliable and comparable. Moreover, the technique is easily applicable, robust, and ideal for routine experiments or for larger scale analyses.

**Electronic supplementary material:**

The online version of this article (doi:10.1186/s13007-016-0149-z) contains supplementary material, which is available to authorized users.

## Background

Reliable methods to assess disease development are of utmost importance when studying plant pathogen interactions in vivo, either to determine plant resistance towards a pathogen or to estimate the aggressiveness of a particular pathogenic strain.

The interaction of Arabidopsis and *Pseudomonas syringae* is a widely used pathosystem to elucidate various aspects of plant-bacterial interactions. In particular, *P. syringae* pathovar *tomato* strain DC3000 (*Pst* DC3000) has been intensely used for numerous molecular investigations to determine how bacterial virulence is established and how host defense responses are activated [1]. Next to the visual evaluation of disease symptoms for resistance or susceptibility of a plant, the plate counting method [2] has been routinely employed to quantify bacterial growth within the host tissue. During this procedure bacteria are re-isolated from leaves and plated on appropriate media in a dilution series to ultimately determine colony forming units per centimeter-squared (cfu/cm^2^). With experienced handling, the method gives an accurate evaluation of the original bacterial load in the plant but it is also quite labor intensive, requires a good number of replicates as well as a well-defined sampling approach since bacterial growth is not always homogeneous within the entire sampled plant tissue. Furthermore, harvested samples need to be directly processed and cannot be stored, which limits the number of samples that can be processed in parallel when performing time course studies or when comparing the pathogenicity of various bacterial strains.

An alternative approach for measuring bacterial growth was proposed in 2008 by using the bioluminescence of a transformed strain of *Pst* DC3000 [3]. The method allows a quick quantification of bacteria and enables high-throughput assays or large-scale quantitative screens. However, the transformation of each bacterial strain and/or mutant with the *luxCDABE* operon from *Photorhabdus luminescens* is necessary to dissect a given plant defense response [3].

The quantification of *Pst* by highly sensitive DNA-based methods like quantitative real-time PCR (qRT-PCR) has been reported by Brouwer et al. [4]. Besides *Pst,* the oomycete pathogen *Hyaloperonospora arabidopsidis*, the necrotrophic fungi *Alternaria brassicicola* and *Botrytis cinerea,* and the bacteria *Erwinia carotovora* were analyzed by the PCR based method. However, normalization of pathogenic DNA in relation to plant biomass was not taken into account. Thus, the previous study provided a solid basis for qRT-PCR based pathogen detection but did not provide full evidence for being an alternative reliable method for the assessment of pathogenic load within the host tissue.

For several pathogens like *Golovinomyces orontii*, *Coletotrichum higginsianum*, *H. arabidopsidis*, *B. cinerea*, and *A. brassicicola* DNA-based methods have now been developed and further optimized to achieve precise measurements for pathogenic growth in *Arabidopsis thaliana* [5–8]. In the case of *Pst* the plate counting assay however has remained the method of choice despite certain disadvantages as indicated above.

Here we report the optimization for qRT-PCR based analysis of *Pst* quantification and its qualitative comparison to the plate counting assay. We show that this DNA-based method can be applied for all general *P. syringae* assays including several *Pseudomonas* strains.

## Results

### DNA-based analysis

An accurate qRT-PCR requires robust primers that efficiently amplify a defined target DNA sequence. We adopted the *oprF* primer pair for a specific DNA region of *Pst* from Brouwer et al. (2003) and ran a nucleotide blast of the primers to the NCBI *Pseudomonas* database. This revealed that these primers are equally suitable to detect several *Pseudomonas* strains relevant for plant studies. These include among others; the common bean pathogen *P. syringae* pv. *phaseolicola* [9], *Pseudomonas cichorii* that infects eggplant, lettuce and tomato [10, 11], *Pseudomonas putida* and *Pseudomonas fluorescens* which are two well studied plant-beneficial microorganisms [12].

In order to guarantee the amplification of a specific DNA region of *Pst* for the quantification of bacterial biomass by using these *oprF* primers, DNA was extracted from pure bacterial cultures of *Pst* DC3000, *Agrobacterium tumefaciens*, *Escherichia coli*, and from germ-free as well as from uninfected and *Pst* DC3000 infected Arabidopsis Col-0 plants ( Additional file 1: Fig. S1). The initial experiment was run with 46 ng DNA for each technical replicate. A specific amplification of the *oprF* PCR product could only be observed for the samples that contained *Pst* DNA (*Pst* DC3000 culture and *Pst* DC3000 infected Arabidopsis). For the other samples and the water control an accumulation of DNA products could only be observed at late time points of the PCR reaction (>30 cycles), yielding an unspecific product.

**Fig. 1:**
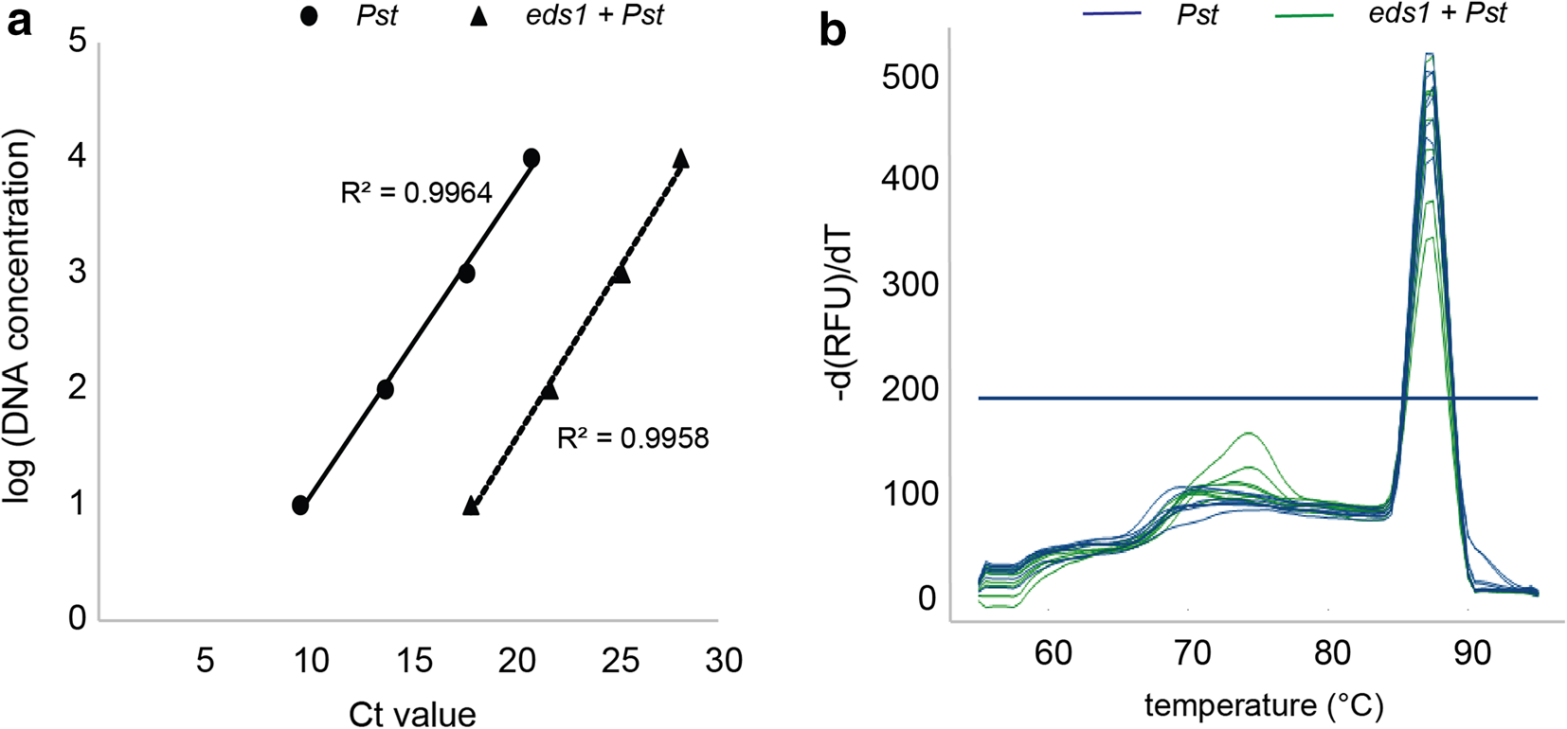
Primer validation for biomass quantification of *Pseudomonas syringae*. **a** The primer efficiency for the PCR quantification of the *oprF* gene from *Pseudomonas syringae* was determined using a dilution series of two different DNA templates. *Closed circles* DNA extracted from a pure *Pst* DC3000 culture; *closed triangles* DNA extracted from *Pst* DC3000 infected *eds1* mutant plants. The respective correlation coefficients (R^2^) are indicated. **b** The PCR products from **a** were used to generate a melting curve analysis. All PCR products melt between 87.0 and 87.5 °C which indicates the breakdown of only one PCR product. A minor peak observed at 75 °C below the indicated melt threshold line very likely represents a contamination that was observed in only two out of eight samples taken from plants

In a second experiment the primer efficiency was tested using a 10-fold dilution series of pure *Pst* DC3000 DNA and *Pst*-infected Arabidopsis DNA. For both DNA samples the primers yielded linear amplification over the range of template concentrations with a correlation coefficient R^2^ > 0.99 (Fig. 1a). Accordingly, the dissociation curves obtained from the PCR products reached their peaks at the same temperature of 87 °C indicating the production of only one specific PCR product during the procedure (Fig. 1b). Taken together, the primer pair is well suited for the quantification of the *Pseudomonas* gene *oprF* even when using low DNA input or samples containing bacterial as well as plant DNA.

### Quantification of *Pst* growth after leaf infiltration

**Fig. 2:**
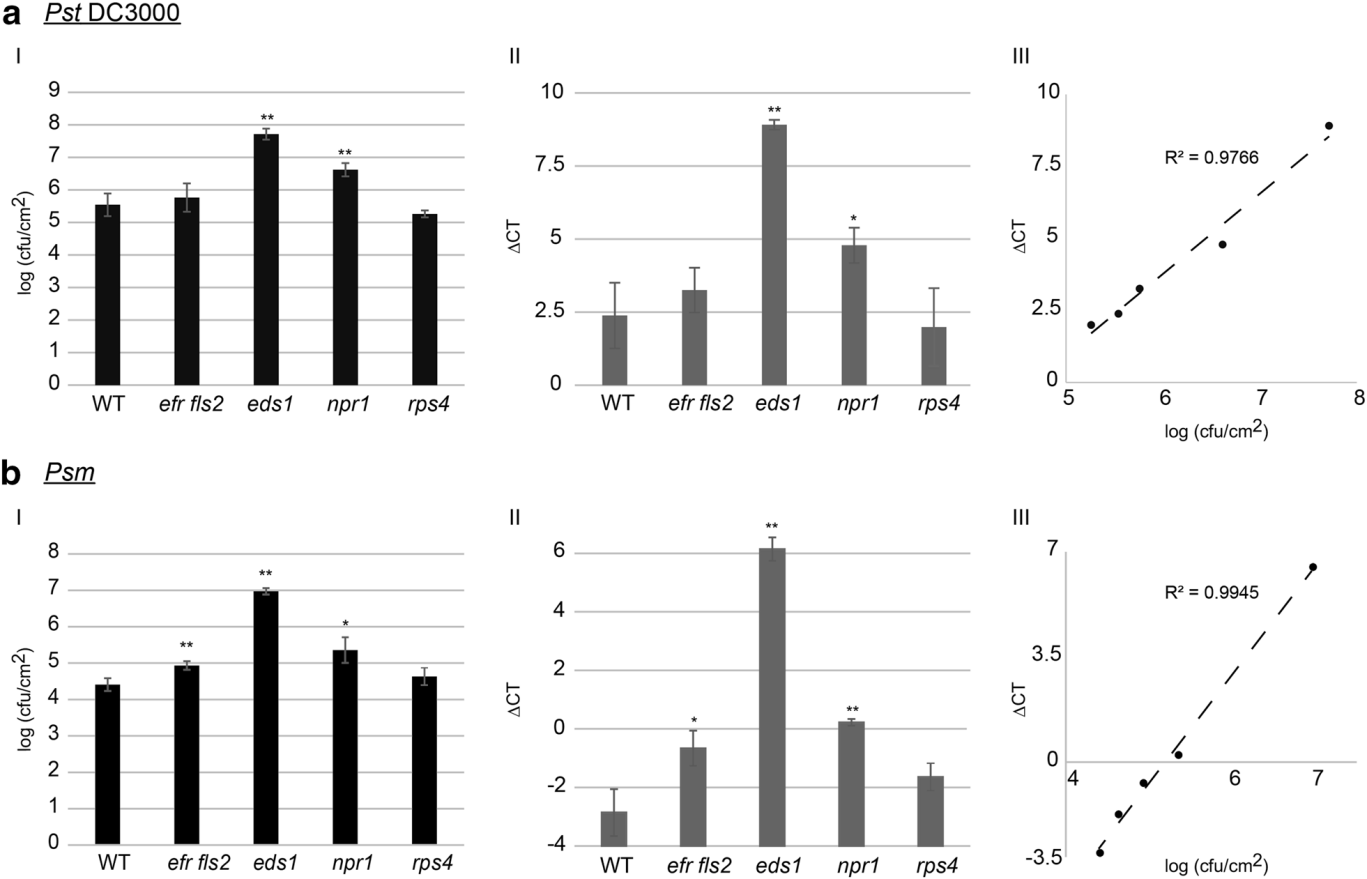
Comparative analysis of the two quantification methods for bacterial growth rates following infiltration of Arabidopsis leaves. **a** The growth of *Pst* DC3000 was determined by the classical colony count quantification method (I) and qRT-PCR-based biomass validation (II). *The error bars* indicate standard deviations of three independent biological replicates. The *stars* indicate statistical significance of the bacterial growth in the indicated mutants in comparison to the bacterial growth in wild-type plants (WT; *t* test: * ≥0.05, ** ≥0.01). The results of the two assays from I and II were plotted against each other (III). The correlation coefficient is indicated (R^2^).** b** The growth of *Pseudomonas syringae* pv. *maculicola* was quantified as for *Pst* DC3000 in A. All experiments were repeated at least three times with comparable results

For direct comparison of the classical plate counting method with qRT-PCR analysis for determination of bacterial growth, Arabidopsis wild-type and four Arabidopsis mutant plants were leaf infiltrated with *Pst* DC3000 (Fig. 2a) or *P. syringae* pv. *maculicola* (Fig. 2b). In order to determine subtle or larger differences in bacterial growth, well described mutants having selected defects in plant defense were chosen for analysis.

As a first layer of defense plants have evolved the ability to sense pathogens by membrane-localized receptors that recognize specific conserved structures unique to the microbe and termed microbe-associated molecular patterns (MAMPs). The receptors FLS2 (Flagellin-sensing 2) and EFR (EF-Tu receptor) have been intensively studied and play an important role for the detection of bacterial pathogens. Loss of these receptors, as in the case of the *efr fls2* double mutant, renders the plants more susceptible to bacterial infections [13–15]. Thus, *efr fls2* mutant plants were selected for further analysis.

The *eds1* mutant was chosen for analysis because EDS1 (enhanced disease susceptibility1) is a key player in basal and effector-triggered immunity specifically mediated by TIR-NB-LRR resistance proteins. EDS1 triggers early plant defense responses including the hypersensitive cell death response and, together with PAD4, further enhances the accumulation of the plant hormone salicylic acid, which is crucial for plant defense against biotrophic and hemi-biotrophic pathogens [16–18]. Several previous studies have demonstrated that loss of *EDS1* leads to enhanced susceptibility towards *Pst* DC3000 [19, 20].

Another central component of plant defense is NPR1 (non-expressor of *PR1*), which modulates the cross-talk of the two defense phytohormones salicylic acid and jasmonic acid and therefore positively contributes to SA-mediated defense against *Pst* DC3000 [21]. We therefore also included the *npr1* mutant in our study.

Host recognition of effector proteins that are released into the plant cell by pathogens to suppress plant basal resistance constitutes the second layer of plant immunity. The resistance protein RPS4 functions as a receptor for the recognition of the bacterial effector AvrRps4. In the absence of RPS4 *Pseudomonas* strains carrying *AvrRps4* (*Pst AvrRps4*) can grow to higher titers *in planta* [22, 23]. Therefore the *rps4* mutant was also included in our analysis.

Employing the classical plate counting assay infiltration of leaves with *Pst* DC3000 resulted in a super-susceptible phenotype on *eds1* and *npr1* plants (Fig. 2aI). In contrast, bacterial growth in leaves of *efr fls2* and *rps4* were lower and reached similar levels as in wild-type plants (Fig. 2aI). The same results were observed by analyzing the bacterial growth by qRT-PCR (Fig. 2aII). Plotting the results of the two experiments in one graph demonstrates a very linear correlation with a coefficient of 0.9766 indicating that the results of the two experiments are highly comparable.

A very similar picture was obtained by analyzing the proliferation of *P. syringae* pv *maculicola*. An enhanced susceptibility could be observed for *efr fls2* and *npr1* mutant plants. In *eds1* mutants the bacterial growth was even more severe whereas *rps4* plants showed comparable bacterial growth levels to wild-type plants (Fig. 2bI). Analyzing the samples by qRT-PCR again showed the same result (Fig. 2bII). Furthermore, correlation analysis of the two experiments showed an almost perfect linear correlation with a coefficient of 0.9945 (Fig. 2bIII).

This comparison therefore showed that monitoring *Pst* bacterial growth in Arabidopsis after leaf infiltration by qRT-PCR analysis was as reliable as the traditional plate counting method.

### Quantification of *Pst* growth after spray inoculation

**Fig. 3:**
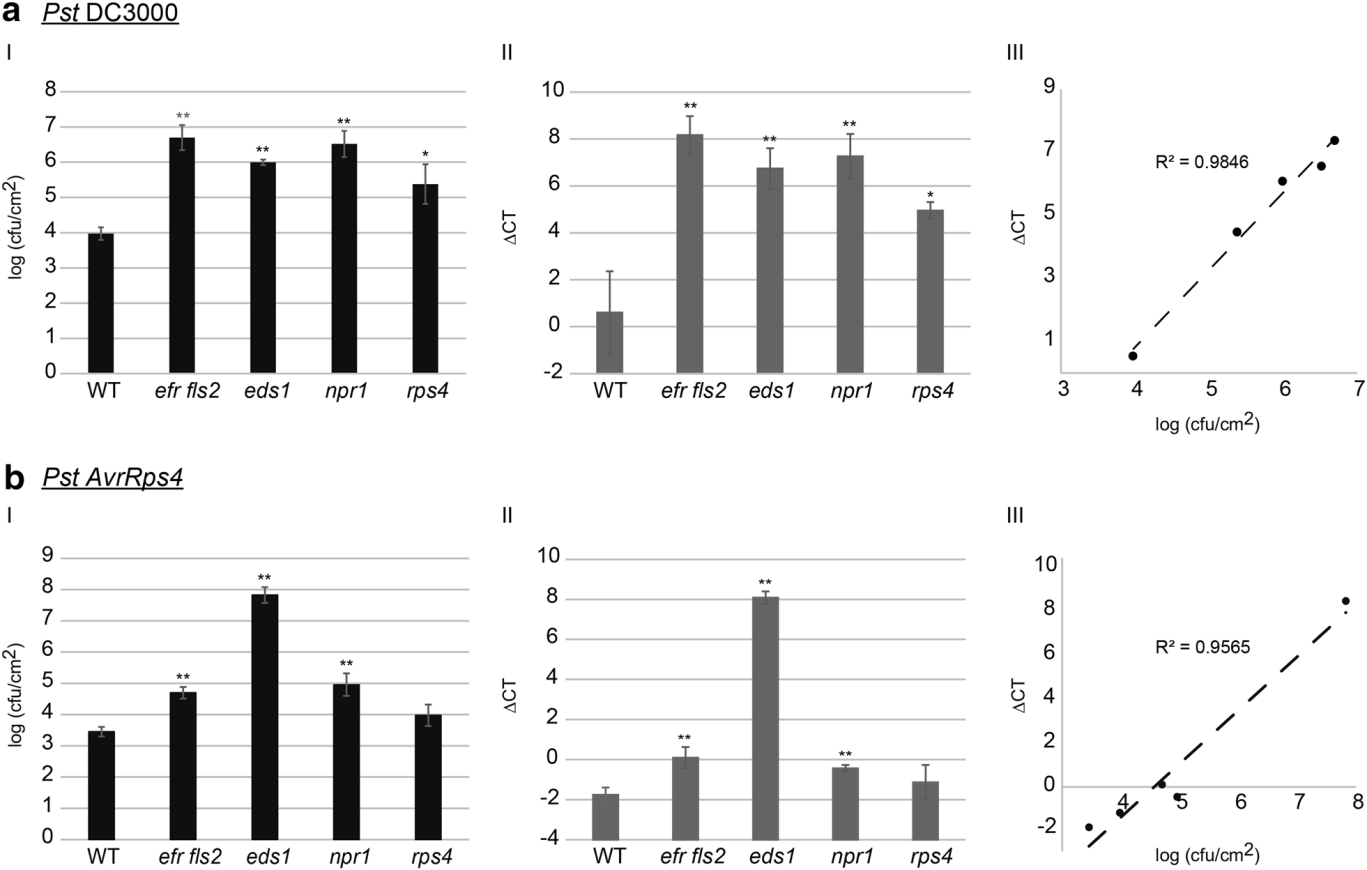
Comparative analysis of the two quantification methods for bacterial growth rates upon spray inoculation of Arabidopsis leaves. **a** The growth of *Pst* DC3000 was determined by the classical colony count quantification method (I) and qRT-PCR-based biomass validation (II). The* error bars* indicate standard deviations of three independent biological replicates. The *stars* indicate statistical significance of the bacterial growth in the indicated mutants in comparison to the bacterial growth in wild-type plants (WT; *t* test: * ≥0.05, ** ≥0.01). The results of the two assays from I and II were plotted against each other (III). The correlation coefficient is indicated (R^2^). **b** The growth of *Pseudomonas syringae* DC3000 *AvrRps4* was quantified as for *Pst* DC3000 in **a**. All experiments were repeated at least three times with comparable results

In order to assess if qRT-PCR can also be used as an alternative method for bacterial quantification upon spray inoculation the above described Arabidopsis genotypes were infected with *Pst* DC3000 and *Pst* DC3000 carrying the effector AvrRps4 (*Pst AvrRps4*) (Fig. 3).

Upon infection by *Pst* DC3000 enhanced bacterial proliferation could be measured by both methods for all four mutants in comparison to the wild-type plants (Fig. 3aI, II). In this case the most susceptible plants were *efr fls2*, followed by *npr1*, *eds1* and finally *rps4*. Exactly the same trend could be observed for both applied methods and is also well reflected in the scatter plot by a linear correlation with a coefficient of 0.9846 (Fig. 3aIII). However, it should be noted that enhanced bacterial growth in *rps4* in comparison to wild-type plants could not be detected in all four independent repetitions.

In the case of spray inoculation with *Pst AvrRps4* an almost linear correlation (R^2^ = 0.9565) could also be observed when the results of the two different methods were plotted against each other (Fig. 3bI, bII, bIII). Wild-type and *rps4* plants allow very little growth of the bacteria. For the mutants *efr fls2* and *npr1* elevated levels of bacteria can be detected in comparison to wild-type plants, whereas highly susceptible *eds1* plants showed highest bacterial titers.

Taken together, similar to the leaf infiltration experiments, bacterial growth assays upon spray inoculation analyzed by qRT-PCR provide an alternative reliable method to plate counting with comparable accuracy.

### Bacterial quantification over time

**Fig. 4:**
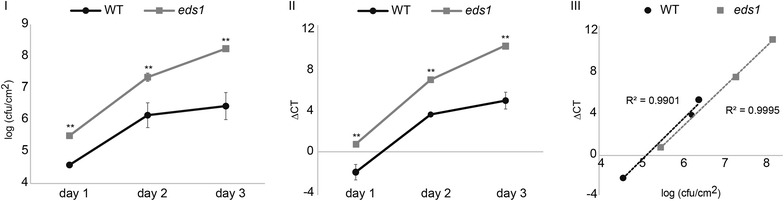
Comparative analysis of the two quantification methods for bacterial growth rates over a time course of infection. The growth of *Pst* DC3000 was determined by the classical colony count quantification method (I) and qRT-PCR-based biomass validation (II) over a time course of three days. The *error bars* indicate standard deviations of three independent biological replicates. The *stars* indicate statistical significance of the bacterial growth in the *eds1* mutant compared to the bacterial growth in wild-type plants (WT; *t* test: * ≥0.05, ** ≥0.01). The results of the two assays from I and II were plotted against each other (III). The correlation coefficients are indicated (R^2^). All experiments were repeated at least three times with comparable results

The quantification of bacterial proliferation over days is an often applied method for demonstration of differences in resistance or susceptibility at a certain time point. To demonstrate that qRT-PCR based quantification of *P. syringae* is also suitable for temporal studies, samples of wild-type and *eds1* plants were taken at one, two and three days after inoculation with *Pst* DC3000 by infiltration and analyzed by plate counting and qRT-PCR (Fig. 4I, II). Both applied methods yielded qualitatively to the same results. As observed in Fig. 2 *eds1* plants are more susceptible than wild-type plants towards *Pst* DC3000 resulting in higher bacterial growth. The difference in bacterial titer can already be observed one day after inoculation and becomes more prominent at day three where bacterial proliferation starts to level off in wild-type plants but continues to increase in *eds1* plants.

Plotting the data of both analyses in one graph results in two almost perfect linear correlations for the two genotypes with coefficients >0.99, indicating that both methods are highly comparable (Fig. 4III).

## Discussion

The quantification of bacterial growth is indispensable for analyzing the interaction between *P. syringae* and *Arabidopsis thaliana*. Although the currently employed plate counting method is highly reliable, this procedure has several disadvantages. Most detrimental is the need of direct processing of the harvested samples excluding the possibility of analyzing short interval time points. Furthermore, it is not well suited for the quantification at early time points. The samples are taken by punching out leaf discs, which only define a surface area but not the volume of the excised leaf disc. When comparing different mutants or plant ecotypes the leaf morphology can significantly differ. Finally, the method is rather labor-intensive and vulnerable for repetitive technical mistakes for instance during pipetting of the dilution series and during counting of the single independent colonies.

DNA-based quantification of *P. syringae* by qRT-PCR seems to be an obvious alternative approach that has meanwhile been adapted to several other plant microbe interaction studies [4–8]. The observed phenotypes in this work largely confirmed already published results, and this was valid for several immune-compromised mutants and wild-type plants showing subtle and larger differences in bacterial growth. The double mutant *efr fls2* for example exhibited enhanced bacterial growth especially upon spray infection [24]. The *eds1* mutant is highly susceptible to several pathogens including *P. syringae* [25]. In line with these findings *eds1* plants exhibited strong bacterial growth in our assays towards both virulent and avirulent *Pseudomonas* strains independent of the applied infection method. Similarly, enhanced bacterial biomass could be detected in *npr1* plants in all assays in accordance to earlier publications demonstrating enhanced susceptibility of *npr1* plants towards infection of *Pst* DC3000 and *Psm* [26–29]. The resistance protein RPS4 recognizes the bacterial effector protein AvrRps4 and subsequently initiates a resistance response. Lack of the resistance protein RPS4 in the *rps4* mutant has been reported to lead to enhanced bacterial growth of *Pst* DC3000 *AvrRps4* [22]. In our study we could not clearly confirm this reported enhanced susceptibility of *rps4* plants neither by the traditional colony counting method nor by qRT-PCR.

The clear advantage of the DNA-based method over the plate counting assay is the accuracy from very low to high amounts of bacterial biomass *in planta*. The amount of DNA is measured by a calibrated PCR machine instead of by a somewhat subjective counting of independent single bacterial colonies within a small area. Additionally, the actual plant biomass instead of a leaf disc diameter is used for the calculation of bacterial growth. The plant biomass can be defined by the PCR-based method because the analyzed plant specific gene (At4g26410) is a single copy gene. Each plant cell harbors one DNA copy of this gene to which the primer can bind during the first round of PCR. The same holds true for the bacteria specific gene *oprF*. Each copy of *oprF* DNA bound by the primer at the beginning of the reaction is representative of one bacterial cell. By subtracting the Ct value of the *oprF* gene from the Ct value of the plant specific gene (ΔCT), the relative abundance of bacterial cells in comparison to the amount of plant cells can be monitored. Moreover, such a DNA-based assay is more suited and reliable in the hands of the less experienced investigator. However, one should be aware that non-degraded DNA of dead/non-viable bacteria will be included in the analysis, which may, under certain cases, lead to a somewhat overestimation of bacterial growth. However, the high comparability of the results presented in this study, which relies on living bacteria only, indicates that the amount of dead bacteria in the samples is often quite negligible, at least during the time period tested.

Another important advantage of the DNA based approach is the possibility for sample storage, which allows close sampling at various time points. Finally, qRT-PCR can be done quickly for larger amounts of samples by using DNA extraction kits or automatized extractions and PCR plate preparations with robots.

## Conclusions

Here we present the quantification of *Pst* by qRT-PCR as an alternative method for assessing bacterial titers in Arabidopsis in comparison to the traditional plate counting method. From our study we can state that both methods are highly comparable and allow for the same biological conclusions for all experiments and this was supported by robust statistical analysis. However,

qRT-PCR for assessing *Pst* bacterial titers in plants has several advantages over plate counting. It brings together the requirements of sensitivity, accuracy, but also rapidity and simplicity that renders it ideal to be used for routine experiments as well as for larger scale analysis.

## Methods

### Plant material and growth conditions

The *Arabidopsis thaliana* accession Col-0 was used as wild-type for all assays in this work and served as the background of the mutants *efr fls2* [24], *eds1*-*2* [30], *npr1*-*1* [31] and *rps4*-*2* [23].

The plants were grown on soil under 10 h light/ 14 h dark conditions at 22 °C and 65% relative humidity for 4 to 5 weeks. Germ-free Arabidopsis plants were grown in sterile ½ MS liquid media for 10 days.

### *Pseudomonas syringae* infections and bacterial growth assay

For spray infection assays a single bacterial colony was picked from plates and grown over night in NYG liquid media supplied with the selective antibiotics at 28 °C. Cultures were collected, washed and resuspended in sterile 10 mM MgCl_2_ at a concentration of 5 × 10^7^ cfu/ml. 0.03% Silwet L-77 (v/v) were supplied to the suspension before spray inoculation of leaves of intact 4–5 week old Arabidopsis plants.

Bacteria for syringe infiltration assays were grown as described above and diluted in 10 mM MgCl_2_ to a final concentration of 1 × 10^5^ cfu/ml. The suspension was then infiltrated into well-expanded leaves of 4 to 5 week old intact plants.

For the bacterial growth assay 6 leaves of 6 individual plants were collected to constitute one sample of three biological replicates. One leaf disc (4 mm diameter) was taken from each leaf for the classical bacterial growth assay. The remaining leaves were frozen at −80 degree for DNA extraction. The six leaf discs were jointly ground in 10 mM MgCl_2_ and subsequently subjected to a 1:10 dilution series. The samples were plated on NYGA solid medium containing the required antibiotics and incubated at 28 °C for two days before colony forming units were counted. Statistical analysis was performed using a Student`s homoscedastic tow-tailed *t* test.

### DNA extraction

DNA was extracted using the FastDNA SPIN Kit for soil (MP Biomedicals). Bacterial cultures or plant leaves were harvested into tubes provided with metal beads and stored at −80 degree or directly processed according to the manufacturer`s instructions. The DNA concentration was determined by Nanodrop and diluted to 3 ng/µl.

### Quantitative real-time PCR

For qPCR analysis about 33 ng of DNA were mixed with 0.4 mM gene specific primers (bacterial biomass: sense AACTGAAAAACACCTTGGGC, anti-sense CCTGGGTTGTTGAAGTGGTA (NC_004578.1) [4]; plant biomass: *A. thaliana* expressed protein At4g26410, sense GAGCTGAAGTGGCTTCCATGAC, anti-sense GGTCCGACATACCCATGATCC [32]) and the iQ SYBR^®^ Green Supermix (Bio-Rad, Hercules, CA) in a total volume of 25 µL. The method was performed on the iQ5 Multicolor Real-Time PCR Detection System (Bio-Rad) with two technical replicates. The abundance of the bacterial derived PCR product was normalized to the abundance of the plant derived PCR product. Statistical analysis was performed using a Student`s homoscedastic two-tailed *t* test.

## Additional file

**Additional file 1: Figure S1.** Amplification of the *oprF* gene product by qRT-PCR using different DNA inputs from several organisms.

## Abbreviations

*Pst*: *Pseudomonas syringae* pv. *tomato*
qRT-PCR: quantitative real-time polymerase chain reaction
