# Supplementary figures and images for: A DNA-based real-time PCR assay for robust growth quantification of the bacterial pathogen Pseudomonas syringae on Arabidopsis thaliana
Source: Plant Methods. 2016 Nov 21;12:48. doi: 10.1186/s13007-016-0149-z (PMC5117497; doi:10.1186/s13007-016-0149-z)

## Slide 1
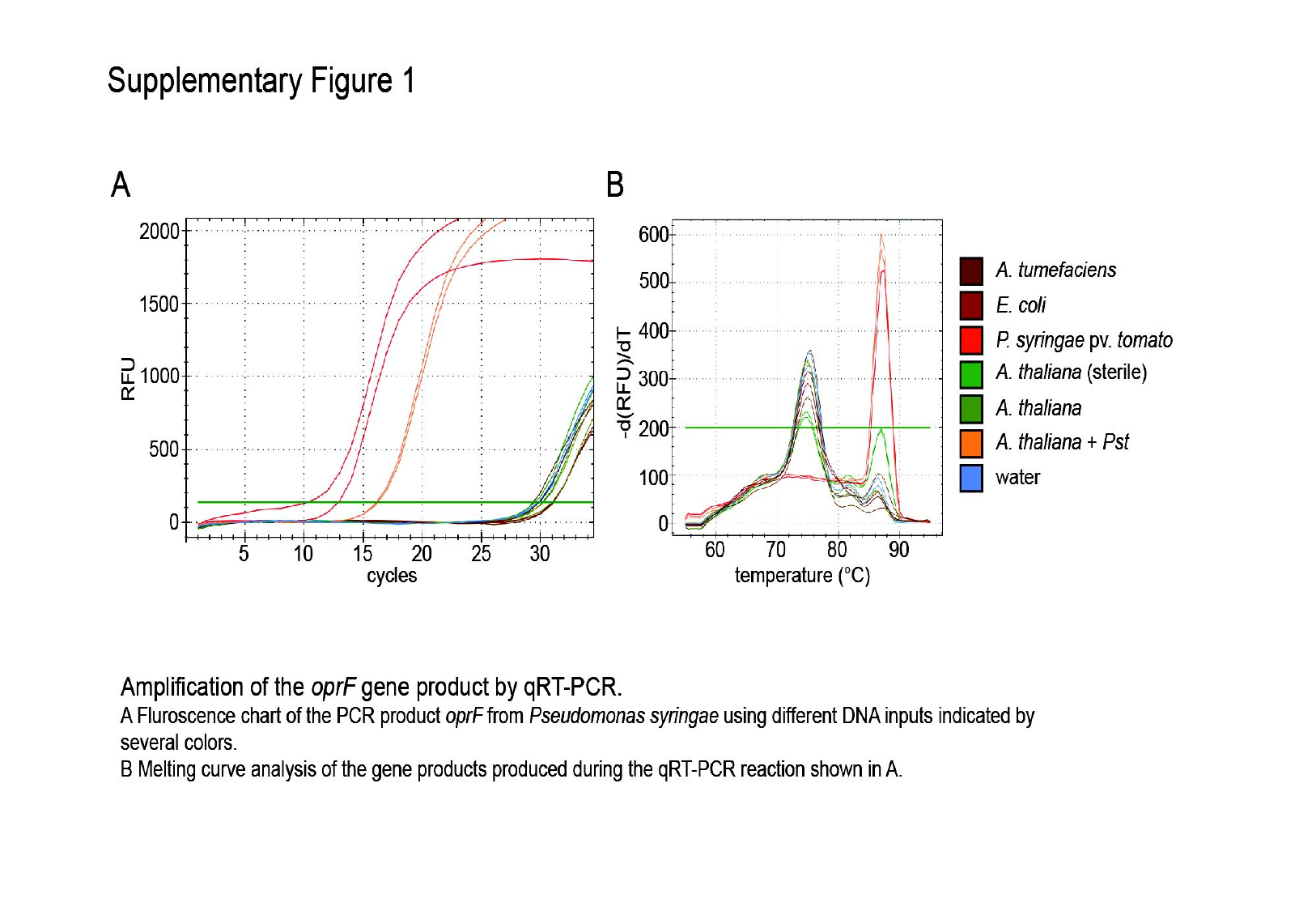

Supplement: Supplementary file 1 — Additional file 1: Figure S1. Amplification of the oprF gene product by qRT-PCR using different DNA inputs from several organisms. [file 13007_2016_149_MOESM1_ESM.pptx]
